# Fecal Microbiota Transplantation in Inflammatory Bowel Disease: A Systematic Review of Efficacy and Safety

**DOI:** 10.7759/cureus.106453

**Published:** 2026-04-05

**Authors:** Hamide Busmail, Sasika Weerakoon, Bethel T Mandefro, Sri Vidya Sundara, Xinyu Lu, Sravanthi Avula, Lubna Mohammed

**Affiliations:** 1 Internal Medicine, California Institute of Behavioral Neurosciences & Psychology, Fairfield, USA; 2 Medicine, California Institute of Behavioral Neurosciences & Psychology, Fairfield, USA; 3 Pediatrics, California Institute of Behavioral Neurosciences & Psychology, Fairfield, USA; 4 Ear, Nose, Throat, Shanghai United Family Pudong Hospital, Shanghai, CHN; 5 Pediatrics, NRI Medical College, Guntur, IND; 6 Principles and Practice of Clinical Research, Harvard T.H. Chan School of Public Health, Boston, USA

**Keywords:** crohn’s disease, fecal microbiota transplantation, inflammatory bowel disease, microbiome, ulcerative colitis

## Abstract

Inflammatory bowel disease (IBD), comprising ulcerative colitis (UC) and Crohn's disease (CD), is a chronic inflammatory condition of the gastrointestinal tract associated with immune dysregulation and alterations in the gut microbiota. Growing evidence suggests that intestinal microbial dysbiosis plays an important role in disease pathogenesis, prompting interest in microbiome-targeted therapies, such as fecal microbiota transplantation (FMT). This systematic review aimed to evaluate the efficacy and safety of FMT in adult patients with IBD. A comprehensive literature search was conducted in PubMed, Embase, Scopus, and the Cochrane Library for studies published between 2020 and 2025 using keywords related to “fecal microbiota transplantation” and “inflammatory bowel disease.” Eligible studies included randomized controlled trials (RCTs), cohort studies, systematic reviews, and meta-analyses involving adult patients with UC or CD. Due to clinical and methodological heterogeneity, a structured narrative synthesis was performed in accordance with Synthesis Without Meta-analysis (SWiM) guidelines. Nine studies comprising 1,847 participants met the inclusion criteria, including five RCTs, two systematic reviews, and two meta-analyses. In patients with UC, clinical remission rates ranged from 32% to 40%, with response rates between 44% and 52%. In CD, remission rates ranged from 24% to 31%, although evidence remained limited and heterogeneous. Multi-donor stool preparations and repeated FMT administrations were associated with improved clinical outcomes compared with single-donor protocols or single-dose protocols. Adverse events occurred in approximately 12-15% of patients and were predominantly mild gastrointestinal symptoms, while serious adverse events were rare (<2%). Current evidence suggests that FMT may induce clinical remission in a subset of patients with UC, while evidence in CD remains less consistent. Larger randomized trials with standardized protocols and long-term follow-up are needed to determine optimal donor selection, dosing strategies, and long-term safety.

## Introduction and background

Inflammatory bowel disease (IBD) is a chronic inflammatory disorder of the gastrointestinal tract characterized by an exaggerated immune response and disruption of the gut microbiota. It primarily includes Crohn's disease (CD) and ulcerative colitis (UC), which differ based on their anatomical distribution and histopathological features [1]. CD involves transmural inflammation that may involve any portion of the gastrointestinal tract, most commonly the terminal ileum and colon, and often presents with skip lesions. In contrast, UC involves continuous mucosal inflammation, beginning in the rectum and extending proximally through the colon [1,2]. IBD typically exhibits a bimodal age distribution, with peak onset between ages 15-30 and a second smaller peak after age 60. Common clinical manifestations include abdominal pain, bloody or non-bloody diarrhea, weight loss, and systemic inflammatory symptoms [3].

The pathogenesis of IBD is complex and multifactorial, involving interactions between genetic susceptibility, environmental exposures, immune dysregulation, and alterations in the gut microbiota [4,5]. Increasing evidence supports a central role for microbial dysbiosis, characterized by reduced microbial diversity, decreased beneficial commensals, and increased pathogenic bacteria [6]. Disruption of the intestinal epithelial barrier further increases mucosal permeability, allowing luminal antigens to trigger chronic inflammation.

Environmental and lifestyle factors, such as antibiotic exposure, Western dietary patterns, and low fiber intake, contribute to microbial imbalance and disease activity [7]. These findings have led to growing interest in therapeutic strategies aimed at restoring microbial homeostasis.

Fecal microbiota transplantation (FMT) has emerged as a microbiome-targeted therapy for IBD. Given the association between dysbiosis and inflammation, microbiota-modulating strategies (probiotics, prebiotics, and FMT) have demonstrated promising clinical outcomes [8]. FMT involves the transfer of processed stool from a healthy donor into the gastrointestinal tract of a recipient with the goal of restoring microbial diversity.

Administration routes include colonoscopy, oral capsules, enemas, or nasoenteric tubes [9]. FMT introduces beneficial microorganisms, such as *Lactobacillus *and *Bifidobacterium *[10]. Donor composition strongly influences efficacy; high *Bacteroides *abundance correlates with positive response, while elevated *Streptococcus *is linked to poor outcomes [11]. Additionally, FMT suppresses pathogenic species, including *Escherichia coli*, *Clostridium difficile*, and biofilm-producing organisms, which improves mucosal barrier function, and modulates host immune responses [10-12].

FMT's Mechanisms 

The therapeutic effects of FMT in IBD are attributed to the restoration of microbial diversity and suppression of pathogenic species. FMT exerts both direct and indirect effects.

Direct effects include competitive inhibition of pathogenic bacteria, enhancement of epithelial regeneration, and stimulation of mucosal immunoglobulin A (IgA) production. Indirect effects involve modulation of host metabolic pathways through microbial metabolites, such as short-chain fatty acids (SCFAs) and bile acids [13].

SCFAs, including butyrate, acetate, and propionate, promote epithelial integrity, strengthen tight junctions, and exert anti-inflammatory effects. Bile acids, metabolized by gut bacteria, regulate immune responses and maintain epithelial homeostasis [14]. Given that approximately 80% of immune cells reside in the gastrointestinal tract, restoration of microbial balance plays a critical role in immune regulation [15].

## Review

Methods 

This systematic review followed the Preferred Reporting Items for Systematic Reviews and Meta-Analyses (PRISMA) and SWiM guidelines. We searched PubMed, Embase, Scopus, and the Cochrane Library. The search strategy combined Medical Subject Headings (MeSH) terms and keywords related to "fecal microbiota transplantation", "inflammatory bowel disease", "ulcerative colitis", and "Crohn's disease". Search operators (AND, OR) were used to refine the search results and maximize the retrieval of relevant studies. 

Eligibility Criteria

Eligible studies included randomized controlled trials (RCTs), cohort studies, systematic reviews, and meta-analyses evaluating fecal microbiota transplantation in adult patients (≥18 years) with UC or CD published between 2020 and 2025. Systematic reviews and meta-analyses were included to provide broader contextual evidence and were analyzed descriptively to support the interpretation of findings from primary clinical studies.

Exclusion Criteria

The following are the excluded studies: non-human studies, pediatric cohorts, non-FMT interventions, or non-IBD indications.

Study Selection and Data Extraction

Two independent reviewers screened studies and extracted data on study design, population, intervention, comparator, outcomes, and adverse events. Discrepancies were resolved by consensus.

Outcomes Valued

Clinical remission: as defined by each study (total Mayo ≤ 2 with no subscore > 1, or Crohn's disease Index (CDAI) < 150). Clinical response: ≥ 3-point Mayo reduction or ≥ 100-point CDAI decrease. Secondary outcomes reviewed included endoscopic remission, steroid-free remission, and safety.

Synthesis Approach

Because systematic reviews and meta-analyses may include overlapping primary studies, their findings were summarized narratively and were not combined quantitatively with the primary randomized trials. Instead, a structured narrative synthesis was performed following SWiM guidelines, focusing on clinical remission, response rates, and adverse events. 

Results 

Study Selection

The literature search identified 459 records across the selected databases. After removal of 16 duplicate records, 443 studies remained for title and abstract screening. Of these, 392 records were excluded because they were not relevant to the research question, including studies not evaluating FMT, studies not involving IBD, nonhuman studies, or review articles without original data.

Fifty-one full-text articles were assessed for eligibility. Following full-text evaluation, 42 studies were excluded for the following reasons: pediatric populations (n = 9), non-FMT interventions (n = 10), non-IBD indications (n = 7), insufficient outcome data (n = 6), non-English publications (n = 5), and conference abstracts without full manuscripts (n = 5).

**Figure 1:**
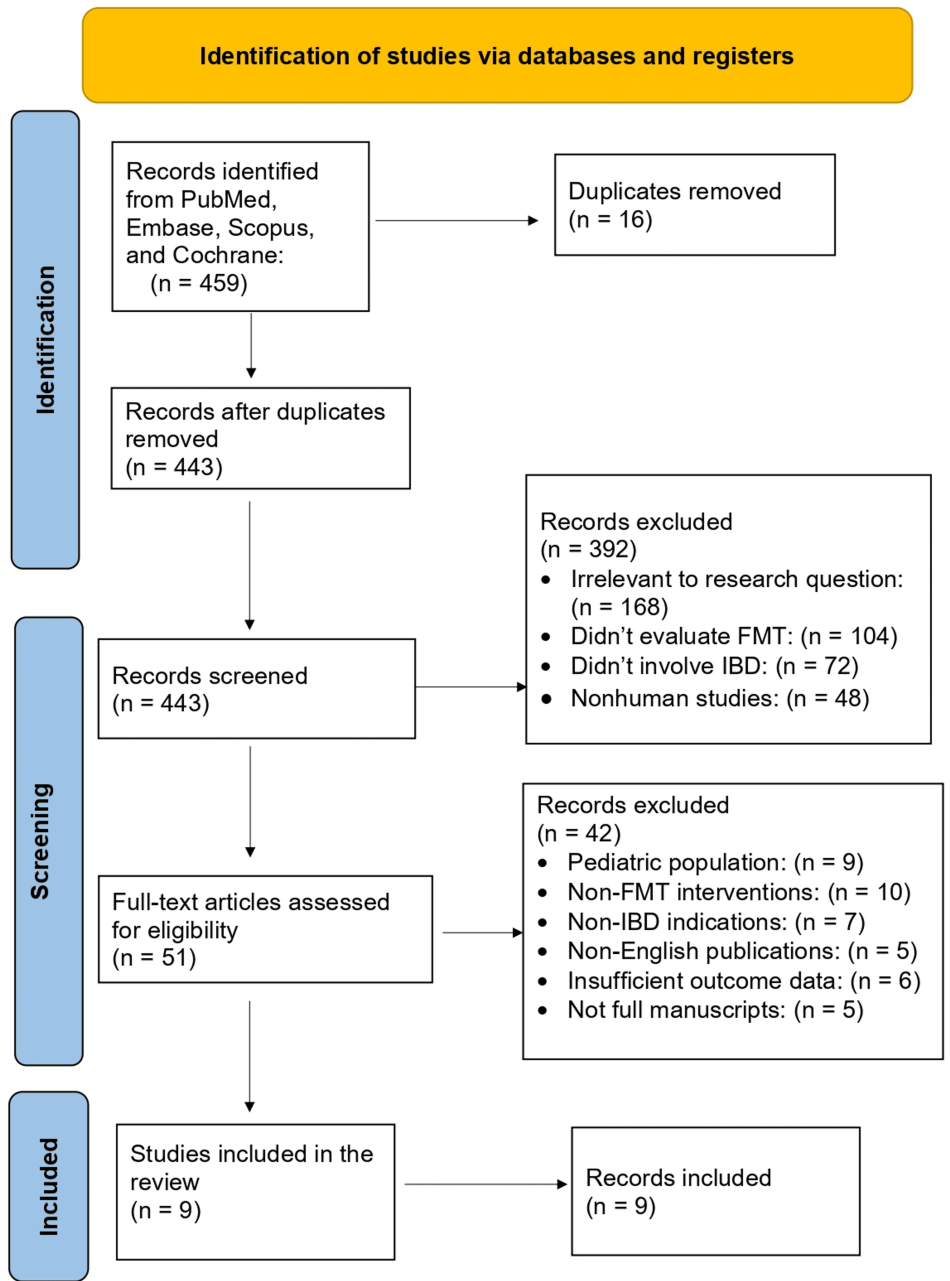
PRISMA flow diagram FMT: fecal microbiota transplantation; IBD: inflammatory bowel disease; PRISMA: Preferred Reporting Items for Systematic Reviews and Meta-Analyses

Ultimately, nine studies met all inclusion criteria, which included five RCTs, two systematic reviews, and two meta-analyses (Figure 1). 

FMT Delivery Variables

Across the studies, several key variables influenced FMT efficacy, such as donor type: related vs unrelated (most studies used universal donors). FMT preparation was either fresh or frozen. Delivery route was via colonoscopy, oral capsules, nasoduodenal tubes, or enemas. The frequency and dosage ranged from single infusions to multiple administrations over several weeks. Some studies included pre-treatment regimens, including corticosteroids or antibiotics [16,17].

Detailed Study Findings

A systematic review composed of 12 studies (550 participants) reported higher clinical remission in UC patients receiving FMT versus controls. Most included cohorts were small and lacked long-term safety evaluation [18]. Early UC treatment with FMT achieved better outcomes, likely due to reversible microbiota disruption [19]. Crothers et al. observed safe long-term oral FMT use but logistical challenges with home storage [20].

A meta-analysis of 18 studies (122 IBD patients) found a 45% clinical remission rate, which decreased to 36.2% when case series were excluded to reduce bias [21]. A trial with 48 UC patients evaluated 54% remission post-FMT versus placebo, but no fecal calprotectin differences after 12 months [22].

Another RCT evaluated four weekly infusions after budesonide pre-treatment; 10 of 24 patients achieved remission, largely donor-dependent [23]. Microbial shifts in *Lactobacillus *and *Ruminococcus *were associated with sustained remission (p < 0.05); repeated dosing at four months improved outcomes [24]. Combined approaches using FMT and probiotics improved remission in UC but showed variable efficacy across other IBD subtypes [25].

Crohn's Disease

Evidence for FMT in CD remains limited. One multi-center RCT showed no endoscopic remission but higher clinical remission rates in FMT versus placebo at eight weeks [26]. Multiple infusions achieved better outcomes than single-dose regimens [27]. Sokol et al. demonstrated improved Crohn's Disease Endoscopic Index of Severity (CDEIS) and reduced C-reactive protein (CRP) post-FMT compared to placebo [28].

Characteristics of the Included Studies

Nine studies comprising 1,847 participants were included (Table 1). Most studies investigated UC, while two examined CD exclusively.

**Table 1:** Summary of the included studies UC: ulcerative colitis; CD: Crohn's disease; FMT: fecal microbiota transplantation; GI: gastrointestinal; IBD: inflammatory bowel disease; RCT: randomized controlled trials; SR: systematic review

Author (Year)	Study Design	Disease Type	Sample Size	FMT Route	Treatment Protocol	Comparator	Clinical outcomes	Adverse Events
Crothers et al. (2021) [16]	RCT	UC	12	Colonoscopy + capsules	Single colonoscopic infusion followed by oral capsules	Placebo	33.3% remission	No serious events
Imdad et al. (2023) [18]	SR	UC/CD	550	Multiple routes	Various FMT protocols	Varied	Higher remission rates vs controls	Mild GI symptoms
Mahmoudi et al. (2022) [20]	SR	IBD	122	Colonoscopy	Single or repeated infusions	None	36.2% remission	Mild GI events
Lahtinen et al. (2023) [21]	RCT	UC	48	Colonoscopy	Single dose infusion	Placebo	54% remission	Mild GI symptoms
van Lingen et al. (2024) [22]	RCT	UC	24	Colonoscopy	Four week infusions + Budesonide	Placebo	42% remission	Mild GI symptoms
Zhang et al. (2024) [24]	Meta-analysis	IBD	-	Multiple	Various protocols	Varied	Overall improved clinical outcomes	Mild GI symptoms
Kao et al. (2024) [26]	RCT	CD	34	Colonoscopy + capsules	Combined colonoscopy and capsule delivery	Placebo	No endoscopic remission	Mild GI symptoms
Fehily et al. (2021) [25]	Meta-analysis + SR	CD/Mixed	-	Multiple	Multiple protocols	-	Varied outcomes	No serious events
Sokol et al. (2020) [28]	RCT	CD	17	Colonoscopy	Single colonoscopic infusion	Placebo	44% remission	None

Efficacy Outcomes

In UC, clinical remission rates reported across individual studies ranged from approximately 32%-40%, with response rates between 44% and 52%. Meta-analyses confirmed comparable remission rates (34%-38%) and moderate heterogeneity. Multi-donor and repeated FMT infusions demonstrated superior efficacy compared with single-donor protocols.

Whereas in CD, reported remission rates ranged between 24% and 31%, while clinical response rates reached up to 45%. Variations in remission criteria across studies (clinical, endoscopic, or biochemical) were documented and summarized in narrative form.

Due to heterogeneity in study designs, outcome definitions, and FMT protocols, results are presented as ranges across studies rather than pooled estimates.

Adverse Events and Safety

Adverse events were reported in 12%-15% of patients, predominantly mild gastrointestinal symptoms, such as bloating, abdominal pain or discomfort, diarrhea, and constipation. Serious adverse events were rarely reported (<2%), with no reported FMT-related deaths [18,29]. Most were transitory and self-resolved; diarrhea occurred in <10%. A risk of infection transmission was also reported, particularly in cases of inadequate donor screening [30].

Risk of Bias Assessment

Most RCTs demonstrated low-to-moderate risk of bias, whereas observation studies were moderate due to confounding and lack of blinding. RCTs were assessed with the Cochrane RoB 2 tool. Systematic reviews were evaluated using AMSTAR-2 (A MeaSurement Tool to Assess systematic Reviews - version 2), which rated moderate quality. Table 2 presents a summary of the risk of bias.

**Table 2:** Risk of bias summary AMSTAR-2: A MeaSurement Tool to Assess systematic Reviews - version 2; RCT: randomized controlled trials; SR: systematic review; RoB 2: Cochrane Risk-of-Bias tool for Randomized Trials

Author (Year)	Study Type	Tool	Overall Risk	Key Concerns
Crothers et al. (2021) [16]	RCT	RoB 2	Moderate	Small sample, randomization unclear
Lahtinen et al. (2023) [21]	RCT	RoB 2	Moderate	Missing data on dropouts
van Lingen et al. (2024) [22]	RCT	RoB 2	Moderate	Small N, incomplete follow-up
Kao et al. (2024) [26]	RCT	RoB 2	Moderate	Reporting bias, early termination
Sokol et al. (2020) [28]	RCT	RoB 2	Moderate	Unblinded assessment
Imdad et al. (2023) [18]	SR	AMSTAR-2	Moderate	Overlap of included studies
Mahmoudi et al. (2022) [20]	SR	AMSTAR-2	Moderate	Publication bias risk
Fehily et al. (2021) [25]	Meta-analysis	AMSTAR-2	Moderate	Heterogeneity, limited stratification
Zhang et al. (2024) [24]	Meta-analysis	AMSTAR-2	Moderate	Selective reporting concerns

Discussion

This systematic review summarizes recent evidence evaluating the efficacy and safety of FMT for the treatment of IBD. Overall, the findings suggest that FMT may induce clinical remission in a subset of patients, particularly those with UC. Across the included studies, remission rates in UC ranged from approximately 32% to 40%, with response rates reaching up to 52%. In contrast, evidence for CD remains more limited, with remission rates ranging from 24% to 31% [18,21,24]. These results are broadly consistent with previous systematic reviews and randomized trials that have demonstrated moderate efficacy of FMT in UC but less consistent outcomes in CD.

Earlier randomized trials have demonstrated the potential of FMT in UC. Moayyedi et al. reported significantly higher remission rates with donor stool compared with placebo, while repeated multi-donor FMT infusions in a trial by Paramsothy et al. achieved improved outcomes, highlighting the benefits of microbial diversity and repeated dosing [31,32]. Evidence in CD is more limited, but Sokol et al. showed improvements in inflammatory markers and endoscopic activity, suggesting that microbiota-based interventions may offer therapeutic potential in selected patients [28].

The greater efficacy observed in UC compared with CD may be explained by differences in disease pathophysiology and anatomical involvement. UC is confined to the colonic mucosa, which allows transplanted microbiota to directly interact with the affected mucosal surface. In contrast, CD involves transmural inflammation and can affect any region of the gastrointestinal tract, which may limit microbial engraftment and therapeutic impact [26,28]. Additionally, disease-specific differences in gut microbiota composition may contribute to variability in treatment response.

Several treatment-related factors appear to influence FMT outcomes. Studies included in this review indicate that repeated administrations and multi-donor stool preparations appear to enhance efficacy compared with single-dose or single-donor protocols. Multi-donor preparations are thought to increase microbial diversity and enhance the likelihood of successful microbiota engraftment. Similarly, studies employing repeated FMT infusions reported improved clinical outcomes, suggesting that sustained microbiome modification may require multiple administrations [17,27]. These findings support the hypothesis that microbiota diversity and donor composition play critical roles in determining treatment success.

The concept of “super-donors,” individuals whose microbiota possess specific compositions that promote higher rates of microbial engraftment and clinical remission, further highlights the importance of donor microbiome composition in determining therapeutic success. Increased abundance of beneficial microorganisms has been associated with improved outcomes, whereas higher levels of potentially pathogenic bacteria may correlate with reduced therapeutic response. These findings highlight the importance of standardized donor screening and microbial profiling in optimizing FMT protocols.

FMT demonstrated a favorable safety profile, with predominantly mild and transient gastrointestinal symptoms. Serious adverse events were rare and occurred in fewer than 2% of cases [29,30]. However, concerns regarding infection transmission highlight the importance of rigorous donor screening.

Limitations

Despite these encouraging findings, several limitations should be considered when interpreting the available evidence. Significant heterogeneity exists across studies with respect to donor selection, stool preparation methods, administration routes, dosing frequency, and patient populations. Additionally, remission and response definitions varied across studies, making direct comparisons challenging. Many studies also included relatively small sample sizes and limited long-term follow-up, which restricts the ability to evaluate sustained efficacy and long-term safety outcomes.

Another important limitation involves potential overlap between primary studies included within systematic reviews and meta-analyses. Although efforts were made to evaluate these sources separately, overlapping data may influence pooled estimates. Furthermore, the lack of standardized FMT protocols across trials underscores the need for large, multicenter RCTs to establish optimal treatment parameters.

Future Direction

Future research should focus on conducting large, multicenter RCTs to establish standardized FMT protocols and determine optimal treatment strategies. Key priorities include identifying ideal donor characteristics, defining effective dosing regimens, and exploring maintenance strategies to sustain microbial engraftment. Advances in microbiome sequencing and metabolomic profiling may facilitate the development of precision-based microbiota therapies tailored to individual patient profiles. Such approaches have the potential to improve clinical outcomes and expand the role of microbiome-directed interventions in the management of IBD.

## Conclusions

FMT represents a promising microbiota-targeted therapy for IBD, particularly UC. Current evidence suggests that FMT may induce remission in a subset of patients, with higher efficacy observed when multi-donor and repeated administration protocols are used. Evidence in CD remains limited and heterogeneous. Overall, FMT appears to be safe, with mostly mild gastrointestinal adverse events. However, larger randomized trials with standardized protocols, optimized donor selection, and long-term follow-up are required to better define its role in routine IBD management.
